# Targeted metabolomics reveals novel diagnostic biomarkers for colorectal cancer

**DOI:** 10.1002/1878-0261.13791

**Published:** 2025-01-03

**Authors:** Zuojian Hu, Fenglin Shen, Yang Liu, Ziqing Zhong, Yongling Chen, Zhiyuan Xia, Cuiju Mo, Hongxiu Yu

**Affiliations:** ^1^ Shanghai Stomatological Hospital & School of Stomatology & Institutes of Biomedical Sciences Fudan University Shanghai China; ^2^ Department of Clinical Laboratory First Affiliated Hospital of Guangxi Medical University Nanning China; ^3^ Department of Colorectal & Anal Surgery First Affiliated Hospital of Guangxi Medical University Nanning China

**Keywords:** carbohydrate metabolites, colorectal cancer, diagnostic model, metabolomics, platelet rich plasma

## Abstract

Colorectal cancer (CRC) is a prevalent malignant tumor worldwide, with a high mortality rate due to its complex etiology and limited early screening techniques. This study aimed to identify potential biomarkers for early detection of CRC utilizing targeted metabolite profiling of platelet‐rich plasma (PRP). Based on multiple reaction monitoring (MRM) mode, liquid chromatography tandem mass spectrometry (LC–MS/MS) analysis identified metabolites in PRP collected from patients with CRC (*n* = 70) and healthy controls (*n* = 30). A total of 302 metabolites were identified and quantified in this study, including various categories such as lipids, lipid mediators, amino acids, and derivatives, organic acids and derivatives, nucleotides and derivatives, alkaloids, carbohydrates, vitamins and derivatives, and others. The differential analysis revealed that five carbohydrates and organic acids (lactose, glycerol‐3‐phosphate, 2−hydroxyglutaric acid, isocitric acid, and citric acid) involved in the carbohydrate metabolism pathway displayed consistent upregulation within PRP derived from patients with CRC. To further validate the abundance of differential metabolites, 10 pairs of CRC tissues, adjacent tissues, and matched PRP were collected. Ultimately, five carbohydrate metabolites were validated in PRP, and compared with carcinoembryonic antigen (CEA) and cancer antigen 19‐9 (CA199), the five carbohydrate metabolites significantly improved the specificity of differentiating patients with CRC from healthy controls. Furthermore, the diagnostic efficacy of the combined five‐carbohydrate metabolite panel was superior to that of individual metabolites, CEA and CA199. The sensitivity, specificity, and AUC of the metabolite panel in distinguishing patients with CRC from healthy controls were 90.00%, 96.67%, and 0.961 (95% CI 0.922–0.998), respectively. Collectively, metabolomics was used to identify and validate differential metabolites in the PRP of CRC, which may serve as potential early screening markers for patients with CRC.

## Graphical abstract

This study employed targeted metabolomic profiling to identify 302 distinct metabolites present in platelet‐rich plasma (PRP), revealing aberrant metabolic profiles amongst individuals diagnosed with colorectal cancer (CRC). Compared to carcinoembryonic antigen (CEA) and cancer antigen 19‐9 (CA199), our metabolite panel showed improved sensitivity, specificity, and AUC. Therefore, this metabolite panel may be a potential tool for early detection of CRC.

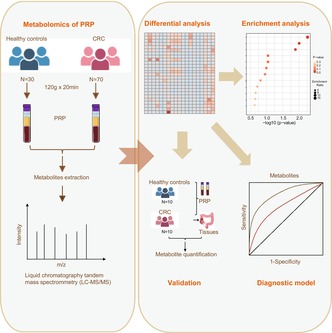

**Abbreviations**
ANOVA: the one‐way analysis of variance
AUC: area under the curve
CA199: cancer antigen 19‐9
CEA: carcinoembryonic antigen
CI: confidence interval
CRC: colorectal cancer
FC: fold change
HPLC: high‐performance liquid chromatography
KEGG: Kyoto Encyclopedia of Genes and Genomes pathway analysis
LC–MS/MS: liquid chromatography tandem mass spectrometry
LTB4: lipoxin B4
MRM: multiple reaction monitoring
mTORC1: mammalian target of rapamycin complex 1
OPLS‐DA: orthogonal partial least squares‐discriminant analysis
PGF2α: prostaglandin F2‐α
PRP: platelet‐rich plasma
PS: Phosphatidylserine
ROC: receiver operating characteristic
TCA: tricarboxylic acid cycle
TNM: tumor node metastasis classification

## 1 Introduction

Colorectal cancer (CRC) is a common type of digestive tract malignancy, characterized by bloody stools, abdominal pain, and anemia [1]. According to the global cancer statistics, CRC has the third‐highest incidence rate and the second‐highest mortality rate amongst all types of cancer [2]. Increased prevalence of CRC within the advanced stage correlates with poor prognosis due to lacking early detection strategies for malignancies [3]. At present, the methods for diagnosing CRC are pathological diagnosis, colonoscopy, and fecal examination in clinical settings. However, these methods have limitations, such as pathological diagnosis and colonoscopy being invasive, expensive, and inconvenient for population screening [2, 4]. Although fecal examination is affordable and less invasive, the accuracy of diagnosis of CRC requires enhancement [2, 3, 4]. Furthermore, a range of hematological biomarkers has been investigated in the diagnosis of CRC. These markers encompass mRNA, circulating cell‐free DNA, microRNAs, long non‐coding RNAs, and proteins [5]. Serum biomarkers such as carcinoembryonic antigen (CEA) and carbohydrate antigen 19‐9 (CA199) have been utilized for phenotyping CRC, but the diagnostic sensitivity of CEA is less than 53%, and the diagnostic sensitivity of CA199 is less than 36% [6, 7, 8]. Therefore, highly sensitive and highly specific hematological markers are urgently required for early screening and clinical diagnosis of CRC.

Cellular metabolic reprogramming, especially changes in energy metabolism, is a hallmark of tumorigenesis [9]. For example, the alterations of metabolic pathways within CRC cells supply essential energy substrates and macronutrients for tumor proliferation [10]. Cancer cells rely on aerobic glycolysis to generate energy and glycolytic intermediates, which serve as precursors for lipids, amino acids, and nucleic acid synthesis [9, 10]. Specific attenuation of aerobic glycolysis cascades has shown potential therapeutic effects, underscoring its significant value for anticancer target development [11]. High‐fat food consumption fosters carcinogenesis through depletion of circulating CD8^+^ T cells, resulting in compromised immunosurveillance capacity against developing lesions [12]. Our previous study has also shown that imbalanced intake of an ω‐6 polyunsaturated fatty acid diet accelerates CRC progression via attenuating intratumoral infiltrations of CD8^+^ T cells coupled with CD4^+^CD39^+^ T cells [13]. Moreover, dysregulated amino acids induced mammalian target of rapamycin complex 1 (mTORC1) activation to drive the development of colon cancers in various mouse models [14]. With the rapid development of omics technology, metabolomics has gained traction as a promising tool for the precocious detection of CRC [15]. The amino acid profile of plasma indicated that tryptophan, sarcosine, and glutamic acid exhibit high sensitivity and specificity in distinguishing CRC patients from healthy controls [3]. Lipidomics has also been used to characterize the lipid distribution of primary and metastatic CRC [16]. Similarly, our previous studies have analyzed sphingolipids and lipid mediators in the serum from patients with CRC and healthy controls [13, 17, 18].

Traditionally, the most frequently employed samples for metabolomics were serum or plasma. In recent years, platelet‐rich plasma (PRP), which contains various bioactive factors, has been widely used in diverse medical fields [19]. Increasing evidence suggests that platelets play an important role in the development of cancers. Different concentrations of PRP in co‐culture with tumor cells were found to affect the biological phenotype of tumor cells [20]. More notably, platelet adhesion facilitates distant metastasis of malignant cells through induction of CD155 expression on the surface of immune checkpoints [21]. Consequently, PRP with higher biological activity could serve as a preferable resource for metabolomics analysis.

In this study, we utilized targeted metabolomics to characterize metabolites in the PRP obtained from patients with CRC and healthy controls. The categories of metabolites identified include lipids, amino acids, carbohydrates and organic acids, nucleotides, alkaloids, vitamins, and others. Differential expression analysis revealed several dysregulated metabolites in PRP from individuals with CRC compared to healthy controls. These differentially expressed metabolites were then validated using both tissue and PRP samples. Subsequently, the diagnostic efficacy of these differential metabolites was evaluated. Overall, this study sought out potential biomarkers associated with CRC via metabolomic characterization of PRP.

## 2 Materials and methods

### 2.1 Clinical sample collection

This study comprised 80 samples of PRP derived from individuals diagnosed with CRC, 40 samples collected from healthy volunteers serving as controls (Ctrl), along with 10 matched sets of primary CRC lesions and uninvolved surrounding tissues harvested during surgical resection procedures. All samples were collected from the First Affiliated Hospital of Guangxi Medical University between May 2023 and July 2024. The inclusion criteria for the samples were patients with initial pathological diagnosis of CRC. The exclusion criteria for the sample are patients with other cancers or chronic systemic diseases. The tumor node metastasis (TNM) staging of CRC follows the standards of the 8th edition of the American Joint Committee on Cancer. The research was carried out in accordance with the standards set by the Declaration of Helsinki and was approved by the Ethics Committee of the First Affiliated Hospital of Guangxi Medical University (2023‐E254‐01 and 2024‐E358‐01). All study participants signed the informed consent according to the institutional guidelines. After collection, whole blood specimens underwent centrifugation under standard laboratory protocols. Samples were spun at 120 g force for 20 min at room temperature. The supernatant (PRP samples) was aliquoted and stored at −80 °C for further analysis.

### 2.2 Metabolite extraction

Owing to the disparate solubility characteristics of water‐soluble and lipid‐soluble molecules, it is essential to employ distinct extraction methodologies to optimize the extraction process. Consequently, each PRP sample was meticulously divided into two equal fractions to facilitate the application of appropriate extraction techniques for each subset.

For water‐soluble metabolites, 1 mL of cold 80% methanol was added with 150 μL of PRP sample, and the mixture was incubated overnight at −30 °C to precipitate the proteins. Tissue samples were weighed for normalization. 1 mL of cold 80% methanol extraction solution was added to the tissue sample along with glass beads, which were then homogenized at 70 Hz using a frozen tissue grinding instrument maintained at −30 °C for 10 min. Then the mixture was also placed at −30 °C for overnight extraction. The following day, the mixture was centrifuged at 16 873 ***g*** for 30 min at 4 °C. The supernatants were further evaporated under vacuum conditions using a speedvac concentrator (Eppendorf, Enfield, CT, USA). Finally, each dry residue was redissolved in 50 μL of 80% methanol for liquid chromatography tandem mass spectrometry (LC–MS/MS) analysis.

For lipid‐soluble metabolites, lipids were extracted based on Folch's method and a modified Bligh‐Dyer's method as previously described [13, 17]. The extract was dried under a stream of nitrogen at room temperature and stored at −80 °C for further analysis.

### 2.3 LC–MS/MS analysis

The identification and quantification of metabolites were performed using LC–MS/MS technology based on multiple reaction monitoring (MRM) mode. All the MRM analysis was performed on a 7500 QTRAP hybrid triple quadrupole/liner ion trap mass spectrometry (AB Sciex LLC, Framingham, MA, USA) interfaced with high‐performance liquid chromatography (HPLC) systems (SHIMADSU, Tokyo, Japan).

For targeted metabolomic analysis, individual samples were fractionated via reverse‐phase HPLC, utilizing a C18 analytical column (Welch Ultimate AQ; 2.1 × 250 mm, particle size = 5 μm) operated at a constant flow rate of 200 μL·min^−1^ over a 20 min linear gradient program. Specifically, the mobile phase consisted initially of solvent A (water containing 0.1% formic acid) held stationary for 1 min followed by a ramping period during which solvent B (acetonitrile containing 0.1% formic acid) increased from 0% up to 90% within 9 min, holding at 90% solution B for 2 min, falling back down to 0% solution B over 0.5 min, followed by 7.5 min at 100% of solvent A. In addition, each sample was also separated on a Waters ACQUITY BEH HILIC (2.1 × 100 mm, 1.7 μm) at a flow rate of 200 μL·min^−1^ using a 15 min gradient, consisting of 80% to 50% solution B (100% ACN) for 3 min, holding at 50% solution B for 7 min, 50% to 80% solution B for 0.1 min, and holding at 20% solution A (10 mm ammonium formate, 0.2% ammonia) for 4.9 min. The LC/MS–MS analysis method for lipid‐soluble metabolites is consistent with our previous research [13, 17]. The transition list and corresponding collision energy were listed in Table S1.

For quantification of five dysregulated metabolites, individual samples were subjected to UPLC utilizing a C18 stationary phase (BEH; Waters Corporation, Milford, MA, USA). The separation process occurred at a constant flow rate of 200 μL·min^−1^ throughout a 15 min linear gradient. The gradient was set as follows: holding at 100% solution A (10 mm ammonium formate, 0.2% ammonia) for 1 min, from 0% to 90% solution B (100% ACN) in 5 min, holding at 90% solution B for 2 min, declining to 0% solution B in 0.1 min, and holding at 100% solution A for 6.9 min.

### 2.4 Cluster analysis and class enrichment analysis

The one‐way analysis of variance (ANOVA) was executed utilizing the *aov* function within the r software (version 4.1.2) to assess the statistical significance of differences amongst the three cohorts: control, stage I–II, and stage III–IV. The metabolites that meet the filter criteria (*P* value < 0.05) were used for subsequent clustering. The expression abundances of these metabolites were subjected to z‐score transformation, followed by the calculation of the mean values for each group. The nbclust package (version 3.0.1) was employed to ascertain the optimal number of clusters. Subsequently, the mfuzz package (version 2.54.0) was utilized for the clustering of metabolites into two distinct groups. Metabolites with a membership value exceeding 0.6 were allocated to their respective clusters. To determine the enrichment level between the metabolite clusters and the metabolite super class, the clusterprofiler package (version 4.2.2) enricher function was applied to perform a hypergeometric test and calculate the corresponding significance values.

### 2.5 Statistical analysis

Skyline analysis software was used to identify peaks of metabolites and quantify peak areas. Data processing and visualization steps were executed through graphpad prism 9 (GraphPad Software, Boston, MA, USA) and spss software (version 26, International Business Machines Corporation, Armonk, NY, USA). The orthogonal partial least squares‐discriminant analysis (OPLS‐DA) score plot, heatmap visualization, and Kyoto Encyclopedia of Genes and Genomes (KEGG) pathway enrichment analysis were conducted utilizing the Metaboanalyst 6.0 platform. In order to compare and contrast differences between groups, we conducted univariate analyses based upon parametric (Student's *t*‐test) or non‐parametric tests (Chi‐Square Test or Mann–Whitney *U* Test). To assess diagnostic efficacy associated with potential biomarkers identified via metabolic profiling techniques, sensitivity, specificity, receiver operating characteristic (ROC) curve, and area under the curve (AUC) were calculated through medcalc software (version 22, MedCalc Software Ltd, Belgium). A Logistic regression model was employed for the classification of CRC patients (*N* = 70) and healthy control (*N* = 30) PRP samples. The model was trained on the expression data of five carbohydrate metabolites. To optimize the model, the entire dataset was subjected to 5‐fold cross‐validation for hyperparameter tuning. The *StratifiedKFold* function was utilized to partition the data into training and validation data. The *grid_search* function was used to tune the hyperparameters. The model (C: 0.01; max_iter: 10000; penalty: l2; solver: lbfgs) was used to fit the whole dataset. Data are expressed as mean ± SEM. *P* value < 0.05 (two‐tailed) was considered statistically significant. For multiple hypothesis testing, the R function *P.adjust* was used to calculate the BH‐adjusted *P* value.

## 3 Results

### 3.1 The clinical characteristics of individuals diagnosed with CRC (CRC group) and healthy controls (Ctrl group)

The clinical characteristics of both the CRC group and the Ctrl group are summarized in Table 1. There was no statistically significant difference in gender (*P* = 1.000) and age (*P* = 0.218) between the two groups. The TNM staging was utilized to categorize individuals affected with CRC according to disease severity levels ranging from stage I through IV. Specifically, the CRC group consisted primarily of patients classified at stage I (*n* = 12), stage II (*n* = 23), stage III (*n* = 18), and stage IV (*n* = 17). The CRC cohort comprised seven cases of T1, seven cases of T2, 48 cases of T3, and eight cases of T4 based on the degree of tumor invasion. Amongst 70 patients with CRC, 34 cases had lymph node metastasis, and 17 cases experienced distant tumor metastasis. CEA and CA199 are known common biomarkers for auxiliary diagnosis of CRC. In the present study, both CEA (*P* = 0.000) and CA199 (*P* = 0.026) were significantly higher in patients with CRC than in healthy controls. The detailed metabolite profiles and clinical information for each individual participant were shown in Table S2.

**Table 1:** Clinical characteristics of colorectal cancer (CRC) group and Ctrl group. CA199, cancer antigen 19‐9; CEA, carcinoembryonic antigen; TNM, tumor node metastasis.

Parameters	CRC (*N* = 70)	Healthy controls (*N* = 30)	*P* value
Age (mean, SD)	60.58 ± 13.13	57.37 ± 8.28	0.218
Gender			
Male	42	18	1.000
Female	28	12	
TNM stage			
I	12		
II	23		
III	18		
IV	17		
Tumor invasion			
T1	7		
T2	7		
T3	48		
T4	8		
Lymph node metastasis			
N0	36		
N1	19		
N2	15		
Distant metastasis			
M0	53		
M1	17		
CEA (ng·mL^−1^, mean, SD)	55.50 ± 216.47	2.68 ± 2.81	0.000
CA199 (U·mL^−1^, mean, SD)	113.11 ± 712.81	17.83 ± 11.65	0.026

### 3.2 Metabolomic profile in PRP samples

**Fig. 1:**
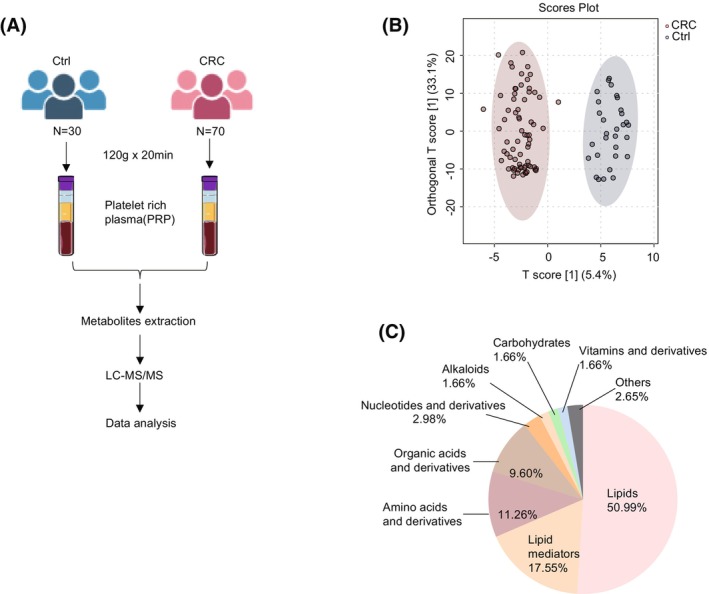
Targeted metabolomics analysis of platelet‐rich plasma (PRP) in colorectal cancer (CRC) patients. (A) Schematic illustration of targeted metabolomics method used to identify characteristic metabolites. (B) Orthogonal partial least squares‐discriminant analysis (OPLS‐DA) score plot for metabolites of PRP samples collected from CRC and healthy controls by liquid chromatography tandem mass spectrometry (LC–MS/MS). (C) Pie chart of metabolite classification identified by metabolomics in CRC and healthy controls.

**Fig. 2:**
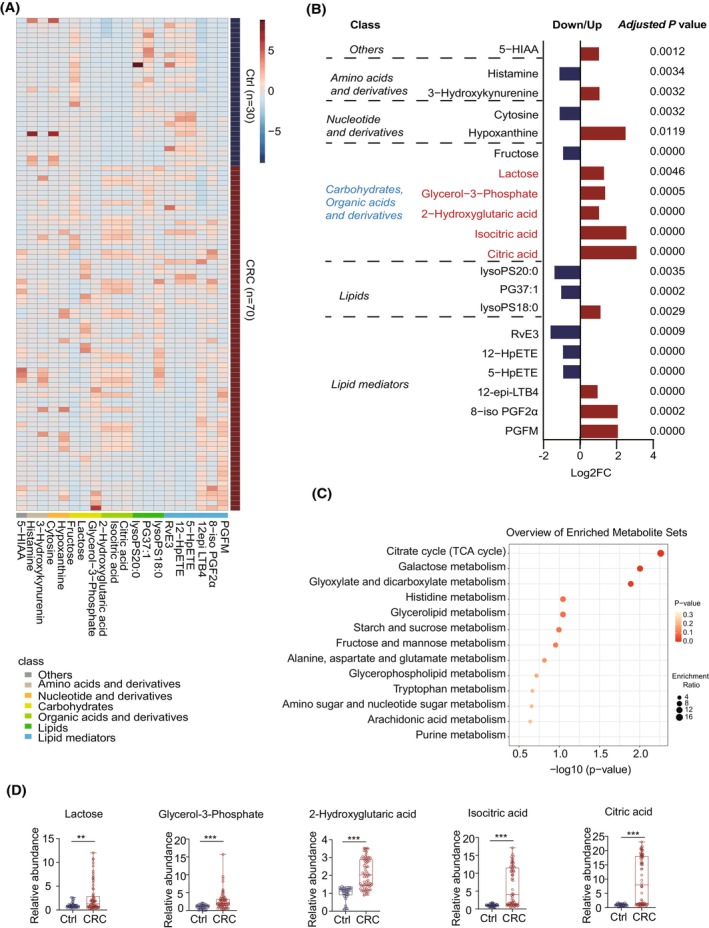
Platelet‐rich plasma (PRP) metabolic profiles in patients with colorectal cancer (CRC) (*n* = 70) and healthy controls (*n* = 30). (A) The heatmap showed the levels of 20 PRP differential metabolites between the CRC group and the control group. (B) Classification and fold changes of differential metabolites between two groups. (C) Pathway enrichment analysis of differential metabolites between two groups. (D) Relative abundance of five upregulated metabolites related to carbohydrate metabolism was observed in CRC patients. **P value < 0.01 and ***P value < 0.001. Error bars indicate SEM. The *P* values indicate results from Student's *t*‐test or BH‐adjusted *P* value. TCA, tricarboxylic acid cycle.

Based on MRM mode, LC–MS/MS technology was used to identify metabolite levels in PRP from 70 patients diagnosed with CRC and 30 healthy controls (Fig. 1A). The supervised OPLS‐DA model effectively differentiated between the CRC group and the Ctrl group according to metabolic profile (Fig. 1B). A total of 302 compounds were identified and quantified in this study, encompassing lipids, lipid mediators, amino acids and derivatives, organic acids and derivatives, nucleotides and derivatives, alkaloids, carbohydrates, vitamins and derivatives, and others (Fig. 1C). Amongst these components, lipids, lipid mediators, amino acids, and organic acids account for a large proportion of overall metabolomic content (Fig. 1C). In order to identify differences in metabolic profiles in PRP obtained from patients diagnosed with CRC and healthy controls, fold change (FC) > 2 or < 0.5 alongside a significance threshold of *P* value < 0.05 were used as the selection criteria. This method resulted in the identification of 20 differential metabolites exhibiting significant alterations associated with CRC. Specifically, two amino acid metabolites, two nucleotide metabolites, three carbohydrate metabolites, three organic acids, three lipids, six lipid mediators, and others were detected at varying degrees of intensity amongst study cohorts (Fig. 2A). The names of differential metabolites were shown in Fig. 2B; five carbohydrates and organic acids (lactose, glycerol‐3‐phosphate, 2−hydroxyglutaric acid, isocitric acid, citric acid) involved in the carbohydrate metabolism pathway showed consistent upregulation in PRP of the CRC group. To better understand the functional roles played by differentially abundant metabolites, Kyoto Encyclopedia of Genes and Genomes (KEGG) pathway analysis was performed. Results revealed three major biological processes emerged as significantly impacted by altered metabolome composition in patients‐derived PRP samples relative to healthy controls, namely the tricarboxylic acid cycle (TCA cycle), galactose metabolism, glyoxylate, and dicarboxylate metabolism (Fig. 2C). Collectively, the above results indicate that abnormal upregulation of carbohydrate metabolism‐related lactose, glycerol‐3‐phosphate, 2−hydroxyglutaric acid, isocitric acid and citric acid, may play an important role in the occurrence and development of CRC (Fig. 2D).

### 3.3 The association between carbohydrate metabolite levels and clinical characteristics in patients with CRC

**Fig. 3:**
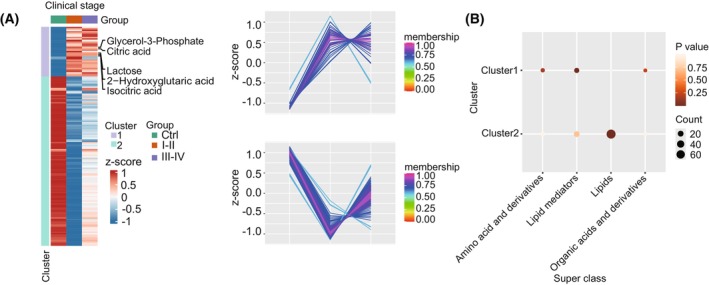
Platelet‐rich plasma (PRP) metabolic profiles unveil metabolic dysregulation in colorectal cancer (CRC) patients. (A) Heatmap of 158 differential metabolites (the one‐way analysis of variance (ANOVA) test, *P* < 0.05) clustered using mFuzz into two discrete significant clusters. (B) Superclass enrichment based on the differential metabolites.

In order to further elucidate the connection between differential metabolites and varying phases of CRC progression, subjects suffering from CRC were stratified according to established TNM staging criteria. A first cohort consisted of patients afflicted with either stage I or II diseases, whereas another consisted of patients experiencing advanced CRC‐labeled stages III and IV, respectively. The hierarchical clustering analysis revealed that the metabolic profiles of the CRC group and the Ctrl group were primarily separated into two distinct clusters. Cluster 1 consisted of metabolites elevated in the CRC group, whereas cluster 2 comprised those decreased in the same group (Fig. 3A). Notably, cluster 1 was enriched in amino acids, lipid mediators, and organic acids, whereas cluster 2 predominantly contained lipids and lipid mediators (Fig. 3B). Furthermore, metabolites involved in carbohydrate metabolism, such as lactose, glycerol‐3‐phosphate, 2‐hydroxyglutarate, isocitrate, and citrate, were found within cluster 1 (Fig. 3A).

**Fig. 4:**
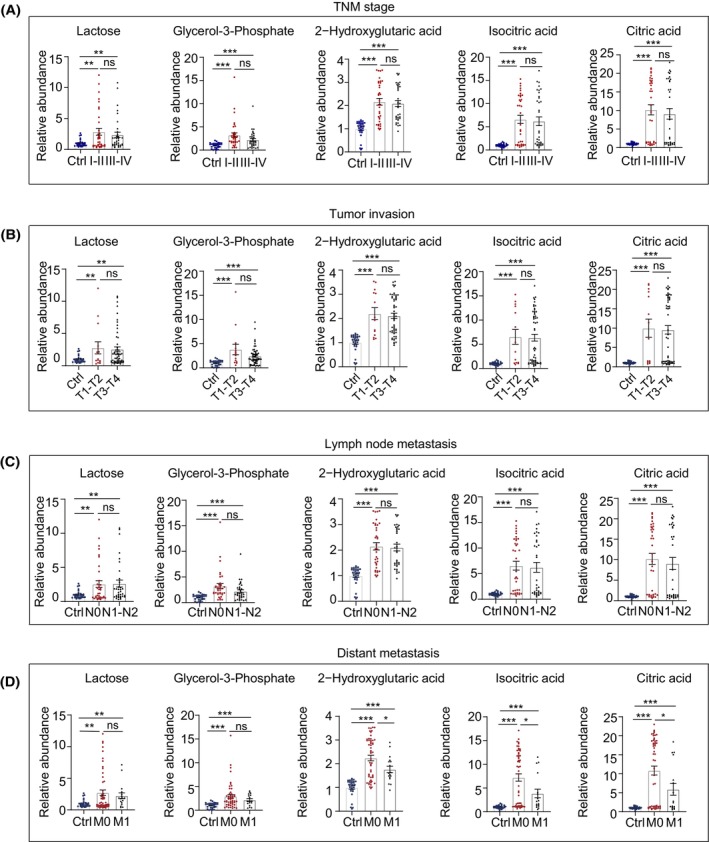
The relative abundance of five carbohydrate metabolites in different stages of colorectal cancer (CRC) progression. (A–D) The relative abundance of lactose, glycerol‐3‐phosphate, 2‐hydroxyglutaric acid, isocitric acid, and citric acid in tumor node metastasis (TNM) staging (A), tumor invasion degree (B), lymph node metastasis (C), or distant metastasis (D) of patients with CRC. *P value < 0.05, **P value < 0.01, ***P value < 0.001, and ns, non‐significant. Error bars indicate SEM. The *P* values indicate results from Student's *t*‐test.

Next, we investigated the abundances of five carbohydrate metabolites across various disease stages. In comparison to the Ctrl group, the concentration of lactose, glycerol‐3‐phosphate, 2‐hydroxyglutarate, isocitrate, and citrate exhibited substantial increases in patients with stage I–II and stage III–IV of CRC (Fig. 4A). Nonetheless, there was no statistical significance in the variation of these metabolite levels between stage I–II versus stage III–IV CRC (Fig. 4A). Similarly, the levels of five carbohydrate metabolites were significantly increased in the T1–T2 and T3–T4 of CRC compared to the healthy controls. Notably, there was no difference in the above five metabolites between T1–T2 and T3–T4 (Fig. 4B). Compared with the CRC group without lymph node metastasis (N0), patients with lymph node metastasis (N1–N2) do not affect the levels of five metabolites (Fig. 4C). In contrast, 2−hydroxyglutaric acid, isocitric acid, and citric acid levels in the CRC group with distant metastases (M1) were significantly lower than in CRC patients without distant metastases (M0) (Fig. 4D).

### 3.4 Tissue metabolomics validation of carbohydrate metabolite contents in PRP

**Fig. 5:**
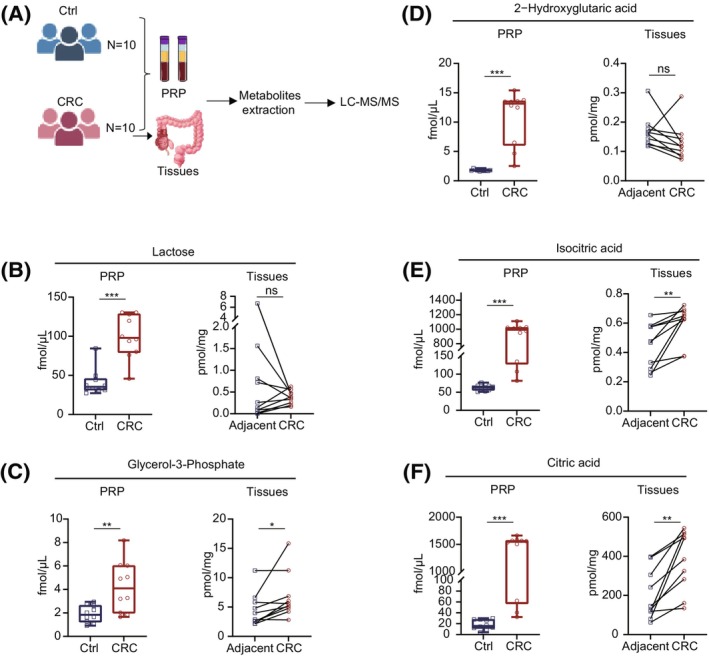
Validation results of five potential metabolic markers in tissues and platelet‐rich plasma (PRP) of colorectal cancer (CRC) patients. (A) Schematic illustration of targeted metabolomics method used to identify metabolites. (B–F) The concentrations of lactose (B), glycerol‐3‐phosphate (C), 2‐hydroxyglutaric acid (D), isocitric acid (E), and citric acid (F) in CRC tissues, adjacent tissues, and PRP. *P value < 0.05, **P value < 0.01, ***P value < 0.001, and ns, non‐significant. Error bars indicate SEM. The *P* values indicate results from Student's *t*‐test. LC‐MS/MS, liquid chromatography tandem mass spectrometry.

Because tissue metabolites can enter the bloodstream via the circulatory system, blood metabolites are subsequently widely synthesized and disseminated throughout the body [22]. Therefore, these metabolites can reflect the metabolic state of the entire organism. To further verify the abundances of lactose, glycerol‐3‐phosphate, 2‐hydroxyglutaric acid, isocitric acid, and citric acid between the CRC group and the Ctrl group, 10 pairs of CRC tissues, adjacent tissues, and PRP were collected (Fig. 5A). Standard curves were generated from varying concentrations of reference compounds to ensure precise measurement of metabolite quantities in both PRP and tissues. Consistent with data shown in Fig. 2D, the contents of lactose, glycerol‐3‐phosphate, 2‐hydroxyglutaric acid, isocitric acid, and citric acid were found to be heightened in PRP extracts isolated from CRC patients as opposed to healthy controls (Fig. 5B–F). Additionally, tissue analysis reveals significant elevations in glycerol‐3‐phosphate, isocitric acid, and citric acid concentrations in CRC tissues over adjacent tissues (Fig. 5C,E,F). These results suggest that these metabolites represent promising diagnostic markers for early detection of CRC.

### 3.5 Diagnostic efficacy analysis of differential metabolites in distinguishing patients with CRC from healthy controls

**Fig. 6:**
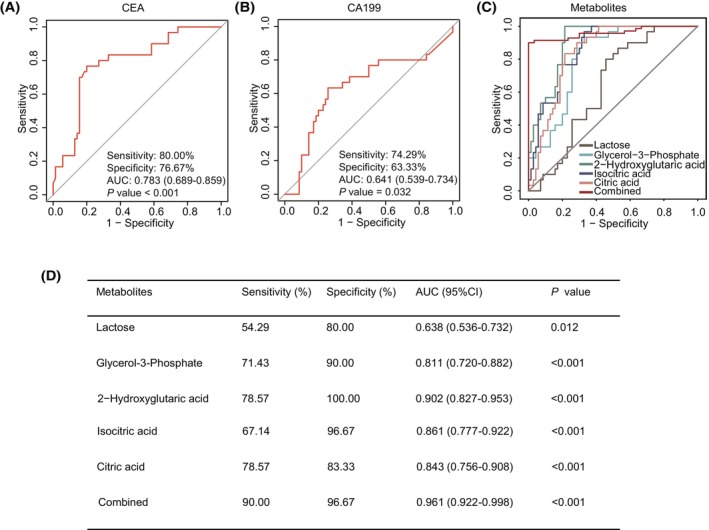
Receiver operating characteristic (ROC) curve analysis of five potential metabolic markers in platelet‐rich plasma (PRP) of colorectal cancer (CRC) patients. (A, B) The diagnostic efficacy of carcinoembryonic antigen (CEA) (A) and cancer antigen 19‐9 (CA199) (B) in distinguishing CRC patients from healthy controls. (C) ROC curve analysis of lactose, glycerol‐3‐phosphate, 2‐hydroxyglutaric acid, isocitric acid, and citric acid in PRP of CRC patients. (D) The sensitivity, specificity, and area under the curve (AUC) of lactose, glycerol‐3‐phosphate, 2‐hydroxyglutaric acid, isocitric acid, and citric acid in distinguishing CRC patients from healthy controls. The *P* values indicate results from the Delong‐method. CI, confidence interval.

The early identification of malignancies is vital for effective therapy and prognostication related to cancers. To confirm the feasibility of using five carbohydrate metabolites to distinguish individuals diagnosed with CRC from healthy controls, metrics such as sensitivity, specificity, and AUC were leveraged to gauge their suitability as reliable diagnostic tools. CEA and CA199 are classic biomarkers for CRC. In this study, the sensitivity, specificity, and AUC of CEA in diagnosing patients with CRC were 80%, 76.67%, and 0.783 (95% CI 0.683–0.859), respectively (Fig. 6A). Similarly, the sensitivity, specificity, and AUC of CA199 in diagnosing patients with CRC were 74.29%, 63.33%, and 0.641 (95% CI 0.539–0.734), respectively (Fig. 6B). Compared with CEA and CA199, the 5 carbohydrate metabolites increased the specificity of distinguishing patients with CRC from healthy controls (Fig. 6C,D). Moreover, apart from lactose, which showed relatively weaker discriminative abilities, the other 4 carbohydrate metabolites displayed impressive ROC curves boasting AUC scores surpassing 0.80 (Fig. 6D). These observations suggest that glycerol‐3‐phosphate, 2‐hydroxyglutaric acid, isocitric acid, and citric acid appear promising as potential metabolic markers for distinguishing patients with CRC from healthy controls.

To improve the diagnostic efficiency of metabolic markers, we used the five carbohydrate metabolites to train a logistic regression model. As shown in Fig. 6A–D, the diagnostic performance of the metabolite panel is greater than that of individual metabolites, CEA and CA199. In particular, the sensitivity, specificity, and AUC of the metabolite panel in distinguishing patients with CRC from healthy controls were 90.00%, 96.67%, and 0.961 (95% CI 0.922–0.998), respectively (Fig. 6D). Collectively, the current findings demonstrate that metabolite panels have better diagnostic efficacy than traditional biomarkers for CRC by providing higher sensitivity and specificity.

## 4 Discussion

Patients with CRC typically exhibit manifestations such as bloody stool, anemia, and abdominal pain preceding diagnostic evaluation and therapeutic intervention [23]. Even though progress has been made toward treating CRC, the mortality rate for CRC remains grim [24]. Therefore, it is imperative to develop screening biomarkers for CRC, which facilitate timely identification and intervention of tumors. The occurrence and development of tumors depend on altered cellular metabolism. Tumor cells acquire energy from abundant nutrients to support unrestrained growth and remodel their metabolic network, typified by the Warburg effect [25]. With the application of vitamins and hormones in clinical laboratories using LC–MS/MS technology, metabolomics and lipidomic analyses have emerged as powerful tools for uncovering novel metabolic biomarkers. Studies have contributed to investigating the diagnosis, prevention, treatment, and pathogenesis of CRC through metabolomics [13, 14, 15, 16, 17, 18]. These studies provided important information for identifying key metabolic pathways and developing cancer metabolomics. However, the detection of metabolites in CRC only focuses on individual classes of metabolites, such as amino acids [3] and lipid profiling [16]. The complexity of human metabolism demands a broader approach encompassing all major nutrient categories, including carbohydrates, amino acids, and lipids.

In recent years, 32 amino acids have been identified in the plasma of patients with CRC based on targeted metabolomics [3]. Meanwhile, lipidomic profiling of plasma‐derived exosomes isolated from CRC patients uncovered 178 distinct lipids [16]. In addition, untargeted metabolomics has become increasingly popular as a tool for characterizing metabolic profiles. A comparative study involving untargeted and targeted quantitative metabolomics approaches was undertaken to characterize the plasma composition of individuals diagnosed with CRC. A total of 147 metabolites belonging to major categories like amino acids, organic acids, and lipids were observed across these groups [26]. Here, in this study, a total of 302 metabolites were identified and quantified in the PRP of patients with CRC, covering various substance categories such as lipids, lipid mediators, amino acids, organic acids, nucleotides, alkaloids, carbohydrates, and vitamins.

Histamine is a crucial amino acid involved in regulating inflammatory responses and tumor progression [27]. Several studies have demonstrated that histamine was decreased in both plasma and urine in patients with CRC, and our results are consistent with previous studies [3, 28]. The downstream product of kynurenine, which is derived from tryptophan metabolism, is 3‐hydroxykynurenine. In CRC, both tryptophan and kynurenine are down‐regulated in the plasma, but there is limited information on how 3‐hydroxykynurenine is affected in this disease [3]. A recent study revealed that low concentrations of 3‐hydroxykynurenine promote tumor growth, while high concentrations of 3‐hydroxykynurenine suppress cell proliferation [29]. Moreover, 3‐hydroxykynurenine has been proven to be a cytotoxic tryptophan metabolite that interrupts the TCA cycle [29]. In this study, 3‐hydroxykynurenine was significantly upregulated in the PRP of patients with CRC compared with healthy controls. In the past, we focused on analyzing the lipidome of serum or tissue in patients with CRC, particularly sphingolipids and lipid mediators [13, 17, 18]. Consistent with the lipidomic of serum and tissues, both RvE3 and 12‐HpETE were also decreased in the PRP of CRC [13, 18]. The eicosanoid compounds lipoxin B4 (LTB4), 12‐epi‐LTB4, and 6‐trans‐12‐epi LTB4 emerge via metabolic transformation of arachidonic acid initiated by lipoxygenase enzymes. LTB4 and 6‐trans‐12‐epi LTB4 were upregulated in the serum or tissue of CRC [13, 18]. In the present study, 12‐epi‐LTB4 was also upregulated in the PRP of patients with CRC compared to healthy controls. Similarly, prostaglandin F2‐α (PGF2α), 8−iso PGF2α, and PGFM were produced by the metabolism of arachidonic acid through cyclooxygenase. In our previous studies, PGF2α was found to be upregulated in both serum and tissue of patients with CRC [18]. Here, in this study, 8−iso PGF2α and PGFM were upregulated in the PRP of patients with CRC compared to healthy controls. Phosphatidylserine (PS) 18:1/18:0 was significantly increased in patients with CRC compared with the healthy controls [16]. LysoPS comprise a class of bioactive molecules generated through enzymatic transformations. This study shows that lysoPS18:0 is increased in the PRP of patients with CRC, while lysoPS20:0 is decreased in the PRP of patients with CRC compared with healthy controls. These results elucidate the disruption of amino acid and lipid metabolism in CRC.

Cancer cells exhibit a distinct metabolic pattern known as the Warburg effect, which involves enhanced glucose consumption and elevated production of lactic acid and ATP [30]. Compared with healthy controls, fructose was downregulated in the PRP of CRC patients, which was consistent with previous studies [31]. Lactose can be decomposed into glucose by lactase, while glucose subsequently produces glycerol‐3‐phosphate numerous biochemical pathways. Glycerol‐3‐phosphate in undergoes further metabolism leading to the generation of acetyl‐coenzyme A, which participates in the TCA cycle [32]. Citric acid and isocitric acid are the main metabolites of the TCA cycle [32]. Moreover, abnormal accumulation of 2‐hydroxyglutarate resulting from isocitrate dehydrogenase mutations serves as a biomarker for various cancers [33]. The upregulation of glucose, glycerol‐3‐phosphate, and citric acid in the serum of patients with CRC has been demonstrated by previous studies [31, 34]. Consistent with the above studies, our findings suggest that various carbohydrate metabolites such as lactose, glycerol‐3‐phosphate, 2‐hydroxyglutaric acid, isocitric acid, and citric acid are upregulated in the PRP of patients with CRC. In addition, the specificity of glycerol‐3‐phosphate, isocitric acid, and citric acid for CRC was validated in CRC tissue, adjacent cancer tissue, and matched PRP. Consequently, these metabolites likely originate from colonic epithelial cells and enter circulation via secretion pathways. However, consistent with previous studies, our analysis did not detect elevated concentrations of lactose and 2‐hydroxyglutaric acid in CRC tissues due to the small number of tissue samples [22].

Patients with advanced CRC may exhibit characteristics of malnutrition and marked decreases in plasma levels of most amino acids [35]. Serum levels of glutamine and histidine were significantly lower in patients with advanced CRC (stages III–IV) than in patients with early CRC (stages I–II) [36]. Similarly, compared to stage I patients, plasma citrulline, histidine, and C17:0 levels were significantly lower amongst stage IV patients [37]. However, another study found no difference in amino acid profiles between early and late stages of CRC [3]. Our findings found no statistically significant difference in PRP for lactose, glycerol‐3‐phosphate, 2‐hydroxyglutaric acid, isocitric acid, and citric acid between stages I–II patients and stages III–IV patients, which are consistent with the amino acid profiles [3]. With the progression of CRC invasion, researchers observed decreasing serum concentration of glutamine [36]. However, glutamine, histidine, and phenylalanine did not correlate with lymph node metastasis [36]. In this study, lactose, glycerol‐3‐phosphate, 2‐hydroxyglutaric acid, isocitric acid, and citric acid could not distinguish the degree of tumor invasion and lymph node metastasis. However, serum levels of glutamine and histidine were significantly reduced in CRC patients with distant metastasis compared to those without distant metastasis [36]. Similarly, mannose and galactose studies suggest a correlation between low serum concentrations of mannose and galactose amongst individuals suffering from liver metastasis resulting from CRC [38]. Consistent with the above studies, our study found that PRP levels of 2‐hydroxyglutaric acid, isocitric acid, and citric acid were decreased in CRC patients with distant metastasis compared to those without distant metastasis [36, 38]. These results elucidate that the progression of CRC was accompanied by changes in metabolite levels.

ROC curve analysis was used to assess the diagnostic efficiency of metabolite markers in differentiating CRC from healthy controls. Compared to traditional biomarkers such as CEA and CA199, lactose, glycerol‐3‐phosphate, 2‐hydroxyglutaric acid, isocitric acid, and citric acid demonstrated higher specificity at identifying CRC. A study showed that the combined diagnostic model of l‐tryptophan, sarcosine, and l‐glutamic acid can significantly improve the diagnostic efficiency of distinguishing CRC patients from healthy controls, with a sensitivity of 0.954 and a specificity of 0.944 [3]. Another study also showed that the combined diagnostic model of l‐phenylalanine, linoleic acid, citric acid, inosine, glycocholic acid, and lysoPC (14:0) can improve the AUC of distinguishing CRC patients from healthy controls [26]. Consistent with the above studies, in our study, the combination of lactose, glycerol‐3‐phosphate, 2‐hydroxyglutaric acid, isocitric acid, and citric acid significantly improved the sensitivity, specificity, and AUC in distinguishing CRC patients from healthy controls [3, 26]. However, this study still has some limitations. Although the differential carbohydrate metabolites were validated in independent PRP and tissues, limited sample size may have hindered the robustness of findings. Thus, future work incorporating larger cohorts across diverse settings would strengthen confidence in conclusions. Importantly, the role of these differential metabolites in the biological function of tumor cells is still unclear. Therefore, it is necessary to evaluate the metabolic functions utilizing biological experiments and explore the pathophysiological basis for CRC development.

## 5 Conclusions

In conclusion, our study employed targeted metabolomic profiling to identify 302 distinct metabolites present in PRP, revealing aberrant metabolic profiles amongst individuals diagnosed with CRC. Importantly, five carbohydrate metabolites (lactose, glycerol‐3‐phosphate, 2‐hydroxyglutaric acid, isocitric acid, and citric acid) were observed to be persistently elevated in the PRP of CRC patients; these findings were further validated in independent PRP and tissue samples. A metabolite panel was constructed using five carbohydrate metabolites to achieve better diagnostic efficacy than individual metabolites. The analysis found that the metabolite panel exhibited high sensitivity and specificity in distinguishing CRC patients from healthy controls. Compared to CEA and CA199, our metabolite panel showed improved sensitivity, specificity, and AUC. Therefore, this metabolite panel may be a potential tool for early detection of CRC.

## Conflict of interest

The authors declare no conflict of interest.

## Author contributions

HY and CM conceived and designed the project; ZH, FS, and YL performed the experiments and analyzed the data; ZZ, YC, and ZX collected the clinical samples; ZH and FS wrote the manuscript, with input from all the authors.

## Supporting information


**Table S1.** The transition list and corresponding collision energy of metabolites.
**Table S2.** The detailed metabolite profiles and clinical information for each individual participant.

## Data Availability

The underlying raw data for this study's findings are accessible upon reasonable request to the corresponding author at hongxiuyu@fudan.edu.cn.
